# Ciprofloxacin induced toxic epidermal necrolysis with cholestatic hepatitis: A case report with literature review and revisit to the Naranjo adverse drug reaction probability scale

**DOI:** 10.1002/ccr3.6766

**Published:** 2022-12-20

**Authors:** Bishal Dhakal, Sagun Dawadi, Laxman Khadka, Sujan Bohara, Shashank Neupane, Priajan Ale Magar, Bishnu Deep Pathak, Binit Upadhaya Regmi

**Affiliations:** ^1^ Nepalese Army Institute of Health Sciences, Sanobharyang Kathmandu Nepal; ^2^ Patan Academy of Health Sciences Kathmandu Nepal; ^3^ Mediciti Hospital Lalitpur Nepal; ^4^ Jibjibe Primary Health Care Center Rasuwa Nepal

**Keywords:** adverse drug reaction, cholestatic hepatitis, ciprofloxacin, Naranjo scale, toxic epidermal necrolysis

## Abstract

Ciprofloxacin, among the many Fluoroquinolones, has been widely used as a broad‐spectrum antibiotic due to its wide range of action and relatively safe adverse effect profile. However, among the cutaneous adverse drug reactions due to Ciprofloxacin, toxic epidermal necrolysis occurring along with cholestatic hepatitis is a rare one. Here, we present a case of a 22‐year‐old male patient who was diagnosed with toxic epidermal necrolysis with cholestatic hepatitis secondary to Ciprofloxacin. Naranjo adverse drug reaction probability scale was used for the causal association.

## 1 INTRODUCTION

Fluoroquinolones (FQs) are an important class of drugs that are commonly prescribed for a variety of clinical conditions since the discovery of the prototype molecule Nalidixic acid in the year 1962.^1^ Depending upon the molecular property and the spectrum of the antibacterial action, they are classified into 1st to 4th generation FQs.^1^, ^2^ Ciprofloxacin belongs to a 2nd generation FQs and is used widely for urinary tract infections, infectious gastroenteritis, musculoskeletal infections, and as a topical antibiotic for bacterial conjunctivitis.^3^ It is a fairly well‐tolerated drug with a good safety profile.^4^, ^5^ However, there have been reports of the adverse drug reaction (ADR) after taking Ciprofloxacin which ranged from an acute purpuric reaction, and idiosyncratic hepatotoxicity to the fatal toxic epidermal necrolysis (TEN) and Steven Johnson syndrome (SJS).^4^, ^5^, ^6^ In this report, we present a case of toxic epidermal necrolysis with cholestatic hepatitis following the intake of Ciprofloxacin as over‐the‐counter medication from a local pharmacy shop. The causal association between the two has been established from the Naranjo method for estimating the probability of ADR.^7^

## 2 CASE PRESENTATION

A 22‐year‐old male patient presented to our hospital with a complaint of a generalized painful fluid‐filled lesion for 15 days, yellowish discoloration of sclera for 7 days, and fever for 1 month following the removal of the implant from his right tibia. According to the patient, he had a road traffic accident 2 years back following which the tibial implant was kept. He had undergone implant removal surgery following which he had a fever secondary to the surgical site infection. At first, he took paracetamol for the fever. As the fever did not subside, he took Ciprofloxacin from a local medical shop. After 2 days of taking Ciprofloxacin, he started having generalized pruritus which progressed to generalized rashes filled with fluids. There were, however, no signs of angioedema with no history of shortness of breath. He also complained of yellowish discoloration of the sclera for 7 days associated with anorexia. However, there was no history of abdominal distention, swelling of lower limbs, hematemesis, melena, and altered sensorium. He denied a history of hypertension, any immunocompromised conditions (HIV or use of steroids/anti‐cancer drugs), and diabetes. He did not consume alcohol or cigarette smoking.

**FIGURE 1:**
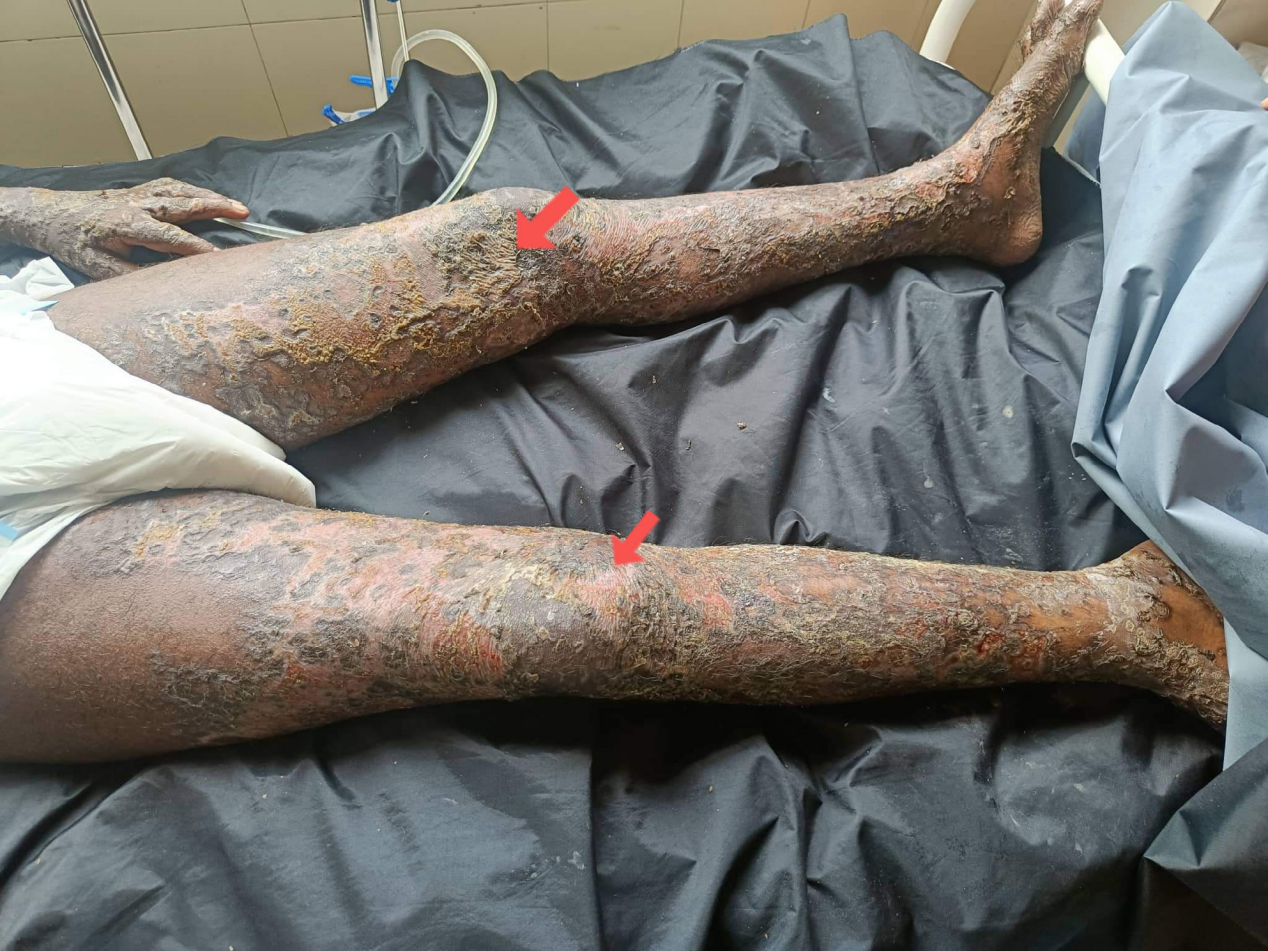
Toxic epidermal necrolysis with multiple generalized vesicles to bullous eruptions (except palms and soles) with crusts (non‐hemorrhagic) (as shown by arrows)

On examination, his vital parameters were stable. Icterus was present. There was no pallor, clubbing, cyanosis, lymphadenopathy, and dehydration. The systemic examinations revealed no abnormalities. Dermatological examinations revealed multiple generalized vesicles to bullous eruptions (except palms and soles) with crusts (non‐hemorrhagic) over bilateral upper and lower extremities and anterior abdomen as shown in Figure 1. Tenderness was present over eruptions along with the post‐inflammatory hyperpigmentation. The mucosal lesions consisted of hemorrhagic crusts in the lips and erosion in the penile area. The laboratory investigations are shown in Table 1.

**TABLE 1:** Laboratory investigations

Laboratory tests	Results	Unit	Reference range
TLC	18,520	Cell/cumm	4000–11,000
DLC			
Neutrophils	64	%	40–70
Lymphocytes	22	%	20–40
Monocytes	2	%	2–10
Eosinophils	12	%	1–6
Basophils	0	%	0–1
Hemoglobin	9.8	g%	13.5–17.5
Platelets	423,000	cells/cumm	150,000–400,000
Red blood cells	3.65	millions/cumm	4.5–5.5
Urea	18	mg/dl	16–49
Creatinine	0.5	mg/dl	0.7–1.3
Sodium	132	mEq/L	135–145
Potassium	4.6	mEq/L	3.5–5.5
SGOT	**283**	U/L	Upto 40
SGPT	**246**	U/L	Upto 41
Alkaline phosphatase	**1390**	U/L	40–130
Total bilirubin	**14**	mg/dl	Upto 1.2
Conjugated bilirubin	**13.7**	mg/dl	0.0–0.4
Total protein	7.9	g/dl	6.4–8.3
Albumin	2.4	g/dl	4.4–7.6
Globulin	5.5	g/dl	2.0–3.5
Prothrombin time test	**32.5**	s	12–17
Prothrombin time control	13	s	
INR	**2.70**		≤1.1

*Note*: Bold values are signifies the ciprofloxacin induced cholestatic hepatitis.

The skin biopsy findings included epidermis showing normal basket weave orthokeratosis with mild acanthosis. There were scattered necrotic keratinocytes, and dermis showed interface dermatitis with melanin pigment incontinence. There were mild to moderate inflammatory infiltrates with lymphocytes and occasional eosinophils in the upper dermis. The impression was consistent with epidermal necrolysis secondary to ciprofloxacin. The ultrasonography (abdomen and pelvis) showed a normal hepatobiliary scan. Based on the clinical manifestation of the patient and the biopsy report, patient was diagnosed with a TEN and drug‐induced cholestatic hepatitis. The causal association between the two was established from the Naranjo method for estimating the probability of ADR with a score of six implicating probable ADR by ciprofloxacin as shown in Table 2.

**TABLE 2:** Naranjo ADR probability score

S. N	Questions	Yes	No	Do not Know
1.	Are there previous conclusive reports on this reaction?	+1	0	0
2.	Did the adverse event appear after the suspected drug was administered?	+2	−1	0
3.	Did the adverse event improve when the drug was discontinued or a specific antagonist was administered?	+1	0	0
4.	Did the adverse event reappear when drug was re‐administered?	+2	−1	0
5.	Are there alternate causes, other than the drug, that could solely have caused the reaction?	−1	+2	0
6.	Did the reaction reappear when a placebo was given?	−1	+1	0
7.	Was the drug detected in the blood (or other fluids) in a concentration known to be toxic?	+1	0	0
8.	Was the reaction more severe when the dose was increased or less severe when the dose was decreased?	+1	0	0
9.	Did the patient have a similar reaction to the same or similar drugs in any previous exposure? Was the adverse event confirmed by objective evidence?	+1	0	0
10.	Was the adverse event confirmed by objective evidence?	+1	0	0
		Total score = +6		

*Note*: Score ≥ 9 = Definitive, 5–8 = Probable, 1–4 = Possible, ≤0 = Doubtful. Yellow shading denotes scoring for our case.

The patient was managed in the intensive care unit (ICU) under the strict aseptic condition with broad‐spectrum antibiotics that consisted of intravenous (IV) Imipenem plus Cilastin (500 mg every 6 h), IV vancomycin (15 mg/kg/dose every 12 h), IV doxycycline (100 mg every 12 h) and supportive management with liquid paraffin, mupirocin, and daily dressing. He was also treated with the tablet cyclosporine (3 mg/kg/day in 2 divided doses) and prednisolone (30 mg daily). The blood culture showed growth of Methicillin‐resistant Staphylococcus aureus (MRSA) and *Klebsiella pneumonia* during the ICU stay and was managed accordingly with the culture sensitivity report. Later, he got improved symptomatically with rearranged liver function test coming back to normal range. He was then discharged with oral medications after 25 days of long hospital stay and advised for follow‐up in medicine and dermatology OPD.

## 3 DISCUSSION

Combined with good oral bioavailability, an embracive antimicrobial action against gram‐positive including pneumococcus, gram‐negative, and anaerobic organisms and a relatively safe adverse effect profile,^1^, ^8^, ^9^ FQs are among the three most commonly prescribed antibiotics group in the United States and Nepal.^10^, ^11^ They are the bactericidal class of antibiotics and work by inhibiting of bacterial DNA synthesis by inhibiting DNA gyrase and topoisomerase IV and enhancing cleavage of DNA in DNA enzyme complex.^12^

Among the FQs, Ciprofloxacin is one of the earlier drugs and has been in use for more than three decades with a good safety profile.^12^ Although there is a paucity of information on the hepatotoxic potential of Ciprofloxacin, it is considered to be significantly low.^13^, ^14^ They have been known to cause a transient elevation of the transaminases which disappears on stopping the drugs over a period of 2–4 weeks while fulminant due to the same has been rarely reported.^6^ In our case, the patient recovered over a period of 2 weeks after Ciprofloxacin was stopped and did not require any specific management for the same.

Cutaneous ADR to Ciprofloxacin has been described to occur at a frequency of 0.5%–3%.^15^ These cutaneous reactions may range from palpable purpura, erythematous rash bulla, and maculopapular rash to erythema multiforme, SJS, and toxic epidermal necrolysis.^5^, ^16^ In a recent systematic review evaluating the cutaneous ADRs of Ciprofloxacin by Kashyap et al., toxic epidermal necrolysis (TEN) was reported in 7 of the 39 included studies followed by fixed drug reaction and drug reaction with eosinophilia and systemic symptoms (DRESS).^5^ TEN is a life‐threatening severe skin condition which starts as constitutional symptoms and is later followed by extensive epidermal necrosis and sloughing, involving more than 30% of the body surface area, sometimes as much as 100% with at least two mucosal involvement.^4^, ^17^, ^18^ This fact is consistent in our patient as he had more than 30% cutaneous involvement and two mucosal surface involvement. TEN is a rare condition with an average occurrence of a 1/million person of which most of the cases are drug‐related.^18^ Among the drugs with a high risk for causing TEN and thus requiring assessment of the risks benefit ratio are Sulphonamides, oxicam NSAIDs, anti‐epileptics like Phenytoin, Phenobarbital, Lamotrigine, and Carbamazepine, while FQ's including Ciprofloxacin are among the agents implicated with appreciably lower risks.^19^ Skin biopsy is done to rule out the other mimics of the disease so that specific treatment for them can be given although the clinical presentation of TEN is unique and flustering.^20^ The usual mimics are Staphylococcal Skin Scalded Syndrome (SSSS), Drug‐Induced Pemphigoid and Pemphigus, Drug triggered Pemphigus, Paraneoplastic pemphigus, Acute Generalized Exanthematous Pustulosis and Acute Graft vs Host disease.^20^ In our case, the biopsy report and clinical presentation of the patient were consistent with the diagnosis of TEN.

Whatever may be the drug causing TEN and as is true for any case of hypersensitivity reaction to a drug, removal of the offending agent is the earliest step in the management of Drug‐Induced TEN. Stopping the offending drug and starting the supportive treatment after admission in the specialized burn/ICU with expertise in handling extensive skin injuries have an important bearing on the overall survival of the patient.^20^ Antibiotic therapy including dosing is guided by the degree of cutaneous involvement and the clinical manifestation of the patient, and the results of culture and sensitivity from the wound swabs and blood.^20^ In our case, the antibiotic therapy was guided by the blood culture report of the patient. The role of systematic steroids in the management of TEN is dubious, and there is a paucity of information on its benefit.^4^ There have been positive results on the efficacy of the intravenous immunoglobulin (IVIG) from different studies thus establishing it as an important additional therapy.^21^ Other adjuvant treatment includes plasmapheresis and immunosuppressant like Cyclosporine, Cyclophosphamide, and TNF‐ alpha inhibitors.^20^ Cyclosporine is given at a dose of 3 mg/kg/day, and its beneficial effect as in our case has been consistently reported in a number of case reports in the past.^20^, ^22^ Despite the care, the mortality with TEN, however, remains as high as 30%.^23^ Necrotizing fasciitis, as a close differential diagnosis, can be ruled out with the absence of local symptoms and signs like severe pain, crepitus, edema, skin necrosis, and foul‐smelling exudates.^24^

In conclusion, Ciprofloxacin though a well‐tolerated drug may have idiosyncratic hypersensitivity reactions in the form of liver injury or life‐threatening TEN. Early recognition and prompt treatment of the patient as in our case help to prevent fatal outcomes. In developing countries like Nepal and other countries in World Health Organization South East‐Asia Region (WHO‐SEARO), the lack of scrutiny of the antibiotics uses and easy availability of over‐the‐counter medications in addition to promoting the bacterial resistance also promotes the occurrence of such complication which may go undetected. Government should employ strict rules on the such prescription of antibiotics and implement an antibiotics stewardship program at each level of health care to prevent such events from happening.

## AUTHOR CONTRIBUTIONS

**Bishal Dhakal:** Conceptualization; methodology; writing – original draft; writing – review and editing. **Sagun Dawadi:** Conceptualization; writing – original draft. **Laxman Khadka:** Investigation; resources. **Sujan Bohara:** Data curation; investigation. **Shashank Neupane:** Investigation; resources. **Priajan Ale Magar:** Data curation; methodology. **Bishnu Deep Pathak:** Supervision; writing – review and editing. **Binit Upadhaya Regmi:** Supervision; writing – review and editing.

## FUNDING INFORMATION

The study did not receive any grant from funding agencies in the public, commercial, or not‐for‐profit sectors.

## CONFLICT OF INTEREST

The authors report no conflicts of interest.

## ETHICAL APPROVAL

This is a case report; therefore, it did not require ethical approval from ethics committee.

## CONSENT

Written informed consent was obtained from the patient for publication of this case report and accompanying images. A copy of the written consent is available for review by the editor‐in‐chief of this journal on request.

## Data Availability

No data were used.
